# The integrated single-cell analysis developed a lactate metabolism-driven signature to improve outcomes and immunotherapy in lung adenocarcinoma

**DOI:** 10.3389/fendo.2023.1154410

**Published:** 2023-03-22

**Authors:** Pengpeng Zhang, Shengbin Pei, Zeitian Gong, Qianhe Ren, Jiaheng Xie, Hong Liu, Wei Wang

**Affiliations:** ^1^ Department of Thoracic Surgery, The First Affiliated Hospital of Nanjing Medical University, Nanjing, China; ^2^ Department of Breast Surgery, The First Affiliated Hospital of Nanjing Medical University, Nanjing, China; ^3^ Department of Burns and Plastic Surgery, The First Affiliated Hospital of Nanjing Medical University, Nanjing, China; ^4^ Department of Thoracic Surgery, The Second Hospital of Nanjing, Nanjing, China

**Keywords:** lung adenocarcinoma, lactate, signature, prognosis, immunotherapy

## Abstract

**Background:**

It has been suggested that lactate metabolism (LM) is crucial for the development of cancer. Using integrated single-cell RNA sequencing (scRNA-seq) analysis, we built predictive models based on LM-related genes (LMRGs) to propose novel targets for the treatment of LUAD patients.

**Methods:**

The most significant genes for LM were identified through the use of the AUCell algorithm and correlation analysis in conjunction with scRNA-seq analysis. To build risk models with superior predictive performance, cox- and lasso-regression were utilized, and these models were validated on multiple external independent datasets. We then explored the differences in the tumor microenvironment (TME), immunotherapy, mutation landscape, and enriched pathways between different risk groups. Finally, cell experiments were conducted to verify the impact of AHSA1 in LUAD.

**Results:**

A total of 590 genes that regulate LM were identified for subsequent analysis. Using cox- and lasso-regression, we constructed a 5-gene signature that can predict the prognosis of patients with LUAD. Notably, we observed differences in TME, immune cell infiltration levels, immune checkpoint levels, and mutation landscapes between different risk groups, which could have important implications for the clinical treatment of LUAD patients.

**Conclusion:**

Based on LMRGs, we constructed a prognostic model that can predict the efficacy of immunotherapy and provide a new direction for treating LUAD.

## 1 Introduction

Lung cancer (LC) is a highly prevalent malignant tumor and the leading cause of cancer-specific deaths worldwide, resulting in over 350 deaths per day in 2022 (1). In the past decade, significant improvements have been made in the science of non-small cell lung cancer (NSCLC), which occupies almost 80% of all LC cases. LUAD is the most common histological subtype of NSCLC. In terms of disease prevention, the wide application of low-dose chest computed tomography has achieved the goal of early detection, greatly reducing all-cause mortality (2, 3). The treatment of LC has also evolved with the generation of several lines of tyrosine kinase inhibitors (TKIs) and immune checkpoint inhibitors (ICIs). Despite that, the 5-year overall survival rate remains poor, ranging from 68% in patients with stage IB to less than 10% in patients with stage IV (4). Thus, it is imperative to explore novel molecular markers for LUAD to improve prognosis.

Since the Warburg effect was proposed in the 1920s, there has been ample evidence that lactic acid plays a critical role in malignant cell proliferation (5). As we know, glucose is the main energy source of tumor cell metabolism. While, due to abnormal metabolic activities, cancer cells desire an excessive quantity of nutrients and oxygen. Based on the Warburg effect, tumor energy metabolism is inclined to anaerobic glycolysis rather than oxidative phosphorylation, even under an aerobic state, which leads to a hypoxic tumor microenvironment (TME) (6). Lactate, the byproduct of glycolysis, is found concentrated in tumor tissue 5-20 times higher than in normal tissue (7). An increased concentration of lactate in the TME is correlated with rapid tumor growth, metastasis, and resurgence, also creating an immunosuppressive TME favorable for a cancer cell to gain immune escape potential (8). Tumor cells may produce lactate and transfer it to surrounding cancer cells, immune cells, and stromal cells, resulting in metabolic reprogramming (9). Thus, lactate plays the role of a mediator between intrinsic metabolism and immunosuppression. Recent studies have identified a number of lactate-metabolizing enzymes that are dysregulated in LUAD, including lactate dehydrogenase A (LDHA), monocarboxylate transporters (MCTs), and lactate oxidase (LOX). Targeting these enzymes with small molecule inhibitors has shown promise as a therapeutic strategy for LUAD (10). Reducing the concentration of lactate by blocking the production pathway of lactate or the transport of lactate has proven to be a promising therapeutic strategy, especially for drug-resistant malignant tumors (11). Although the LMRGs have been proven to perform a critical function in the progression of LUAD in recent years (12, 13), comprehensive analyses of the relationship between LMRGs and the diagnosis, risk stratification, and prognosis of LUAD are urgently needed.

Hence, in the present study, we aimed to screen out the LMRGs in LUAD and elaborate on the role of LMRGs in the TME and prognosis of LUAD. Then, we will establish a signature capable of predicting the prognosis of patients with LUAD on basis of LMRGs. Our research may improve the existing lactate-dependent therapeutic schedule, providing novel insights into prognostic biomarkers and therapeutic targets for LUAD.

## 2 Materials and methods

### 2.1 Data acquisition

In this study, LUAD scRNA-seq data were obtained from the GSE150938 database (https://www.ncbi.nlm.nih.gov/geo/), which consisted of 12 LUAD samples. The training cohort comprised LUAD RNA expression patterns and relevant clinical information from The Cancer Genome Atlas (TCGA) database (https://portal.gdc.cancer.gov/). Additionally, the validation set was obtained from the GSE29016, GSE30219, GSE31210, and GSE42127 GEO expression profiles. To facilitate data comparability, all expression data were converted to transcripts per million (TPM) format. The “sva” R package was used to eliminate the batch effect, and all data were transformed to log2 before analysis. A total of 247 lactate-related metabolic genes (LMRGs) with correlation values greater than 15 were selected from the GeneCards database (https://www.genecards.org/) for further analysis.

### 2.2 scRNA-seq data processing and cell annotation

We validated the scRNA-seq data using the “Seurat” R program. Screening criteria included expressing genes in at least three cells, expressing 200-7000 genes in each cell, and expressing no more than 10% of mitochondrial genes. Finally, 46,286 appropriate cells were identified. The top 3000 highly variable genes were screened using the “FindVariableFeatures” program. The canonical correlation analysis (CCA) function “findintegrationanchors” was used to reduce batch effects that might interfere with downstream analysis. We utilized the “IntegrateData” and “ScaleData” methods to appropriately integrate and expand the data. Anchor points were discovered using principal component analysis (PCA) dimensionality reduction. To locate relevant clusters, the first 20 PC were tested using the t-distribution random neighborhood embedding (t-SNE) technique. We obtained 20 cell clusters by using the “FindNeighbors” and “FindClusters” functions (resolution = 0.8). We assessed cell cycle heterogeneity along cell clusters using cell cycle markers from the “seurat” package. The “CellCycleScoring” program was used to generate cell cycle scores based on the expression of G2/M and S-phase markers. The “FindAllMarkers” program was used to identify differentially expressed genes (DEGs) for each cluster. To select which genes were employed as markers for each cluster, we used a cut-off threshold and modified P< 0.01 and log2 (foldchange) > 0.25 criterion. Cell types were meticulously defined using common marker genes for each cluster. The “AUCell” R program, which analyzes the activity state of gene sets, was used to assign LM activity ratings to each cell. The cells were separated into high- and low-LM-AUC groups based on the median AUC score, and visualization was done with the “ggplot2” R program. We next performed differential analyses to discover DEGs in high- and low-LM-AUC groups, and 440 DEGs were selected for further investigation. Furthermore, we used correlation analysis to look at the genes most connected with LM activity, with the top 150 most associated genes being included for future study. The DEGs and genes discovered through association analysis were the ones that had the greatest effect on LM activity (590 genes in total).

### 2.3 Construction and validation of the risk scoring

We used univariate analysis on the 590 genes that regulated LM activity to find genes that significantly related with patient survival (P< 0.01). Following that, LASSO and multivariate regression analysis were used to further screen for genes and risk coefficients that were highly linked with prognosis. Based on the coefficients revealed by the multivariate analysis, each LUAD patient was assigned a risk score. Patients from the TCGA-LUAD were separated into high- and low-risk groups based on their median risk score. Meanwhile, survival curves were plotted using the Kaplan-Meier technique for prognostic reasons, and log-rank tests were used to establish statistical significance. The prediction model’s effectiveness was evaluated using receiver operating characteristic (ROC) curves; an AUC value of >0.65 indicated outstanding performance. The signature’s prediction capacity was verified in nine distinct GEO datasets using survival analysis and AUC. PCA analysis was used to show the distribution of patients in different risk groups. A similar method was used to validation cohorts.

### 2.4 Nomogram construction

We created a nomogram that used the risk score, age, and pathological stage as independent prognostic criteria to compute the probability of OS at 1-, 3-, and 5- years (14). The receiver operating characteristic (ROC) curve, calibration curve, and concordance index curve were also utilized to evaluate the prediction accuracy of the nomogram. The prognostic significance of risk score clinical characteristics was assessed using stratified analysis (age, pathological T, N stage, and clinical stage).

### 2.5 Mutation landscape

The TCGA database was used to generate gene mutation profiles from LUAD patients, and the “ComplexHeatmap” R package was used to visualize the mutation landscape and immune infiltration scores (15). According to the median risk score and tumor mutational load, TCGA-LUAD patients were separated into four groups (H-TMB+high-risk, H-TMB+low-risk, L-TMB+high-risk, and L-TMB+low-risk), and their survival disparities were compared.

### 2.6 Assessment of immune infiltration

The timer 2.0 database was used to download data from seven different methods that were utilized to determine the degree of immune infiltration in TCGA-LUAD patients. A heatmap graphic was used to show differences in immune infiltration across various risk categories. The “estimate” R program was used to quantify the stromal and immune cell abundance and tumor purity in malignant tumor tissues based on the expression patterns (16). A higher score indicates that there is a greater percentage of TME components.

### 2.7 Immunotherapy comparisons

Immune checkpoints are a group of molecules that are expressed on immunological cells that control the amount of immune activation. They are critical in controlling excessive immunological activation. We evaluated the levels of expression of well-known immune checkpoint genes in both groups (ICGs). Correlations between ICGs expression, model genes, and risk scores were investigated further. The Immunophenoscores (IPS) for LUAD were obtained from the Cancer Immunome Atlas (TCIA) database (17).

### 2.8 Enrichment analysis

The GSVA used the MSigDB signature gene sets “h.all.v7.5.1.symbols.gmt” (https://www.gseamsigdb.org/gsea/msigdb/index.jsp). The GSEABase program was then used to analyze the activity of each gene set in each sample. GSEA was used to identify which signaling pathways and biological activities were enriched in the high- and low-risk groups. ssGSEA was used to determine the enrichment scores of infiltrating immune cells and immunological function.

### 2.9 The Role of AHSA1 in LUAD

Using the timer database, researchers investigated the expression of AHSA1 in pan-cancer. Patients were divided into two groups based on AHSA1 expression to study changes in survival: both high- and low- expression.

### 2.10 Cell lines culture

The Cell Resource Center at Shanghai Life Sciences Institute provided BEAS-2B, A549, and H1299 human LUAD cell lines. These cells were cultured in F12K or RPMI-1640 (Gibco BRL, USA) with 10% FBS, 1% streptomycin, and penicillin (Gibco, Invitrogen, Waltham, MA, USA). Cells were grown at 37°C, 5% CO2, and 95% humidity.

### 2.11 Cell transfection

SIRNAs knocked down AHSA1 (siRNAs). **Supplementary Table S1** included AHSA1 siRNA sequences. In a 6-well plate, cells were plated at 50% confluence and infected with negative control (NC) and knockdown (siAHSA1). All transfections used Lipofectamine 3000 (Invitrogen, USA).

### 2.12 Extraction of RNA and Real-Time PCR (RT-PCR)

Cell lines were TRIzol-extracted for total RNA (15596018, Thermo). Using PrimeScriptTMRT, cDNA was made (R232-01, Vazyme). SYBR Green Master Mix (Q111-02, Vazyme) was used for real-time polymerase chain reaction (RT-PCR), and each mRNA was standardized to GAPDH. Expression levels were counted using 2−-Ct. **Supplementary Table S1** lists all primer sequences from Beijing-based Tsingke Biotech.

### 2.13 Colony formation

We transfected 1x10^3^ cells into each well of a 6-well dish and cultured them for 14 days. The cells were fixed in 4% paraformaldehyde for 15 minutes and then stained with Crystal violet (Solarbio, China).

### 2.14 EdU

After the cells adhered to the side of the 96-well plate, the experiment was performed. Then, the manufacturer’s 5-Ethynyl-2’-deoxyuridine (EdU) test was carried out (Ribobio, China). Cells that were actively dividing were tallied using an inverted microscope.

### 2.15 Wound-healing assay

A cell incubator was used to grow transfected cells in 6-well plates to 95% confluence. Each cultured well was scraped along a single straight line using a sterile 20-L plastic pipette tip, and the scrapings and any loose cells or debris were rinsed away twice with phosphate-buffered saline. Taking pictures of the scratches at 0h and 48h, we next used the Image J program to quantify the breadth of the wounds.

### 2.16 Transwell assay

The transwell test was used to examine the invading and migrating cells. Incubation of treated A549 and H1299 cells (2x10^5^ per well) in 24-well plates began after 12 hours. The cells’ invading and migrating abilities were measured by precoating the top of the plate with matrigel solution (BD Biosciences, USA) or leaving it untreated. The cells on the top surface were removed, while the remaining cells on the bottom were fixed in 4% paraformaldehyde and stained with 0.1% crystal violet (Solarbio, China).

### 2.17 Statistical analysis

Data and statistics were processed in R (version 4.1.3). The experimental data were analyzed using Graphpad and Image J (1.8.0) (version 9.4.0). Kaplan-Meier curves and a log-rank test were used to evaluate the differences in survival times between the two groups (18). The “survminer” R program was used to generate all survival curves. Cox and lasso regression analysis were used to assess risk factors. For visualization, we utilized “ggplot2” program, and for analysis, the R package “survival” was used to calculate both OS and risk scores. It was made using “Pheatmap”, an online heatmap generator. Significant quantitative differences for normally distributed variables were identified using a two-tailed t-test or a one-way analysis of variance. For non-normally distributed data, the significance of any differences was determined using either the Wilcoxon test or the Kruskal-Wallis test. All statistical testing was performed in R. If the number is less than 0.05, it is considered to be statistically significant.

## 3 Results

### 3.1 Analysis process of scRNA-seq

**Figure 1:**
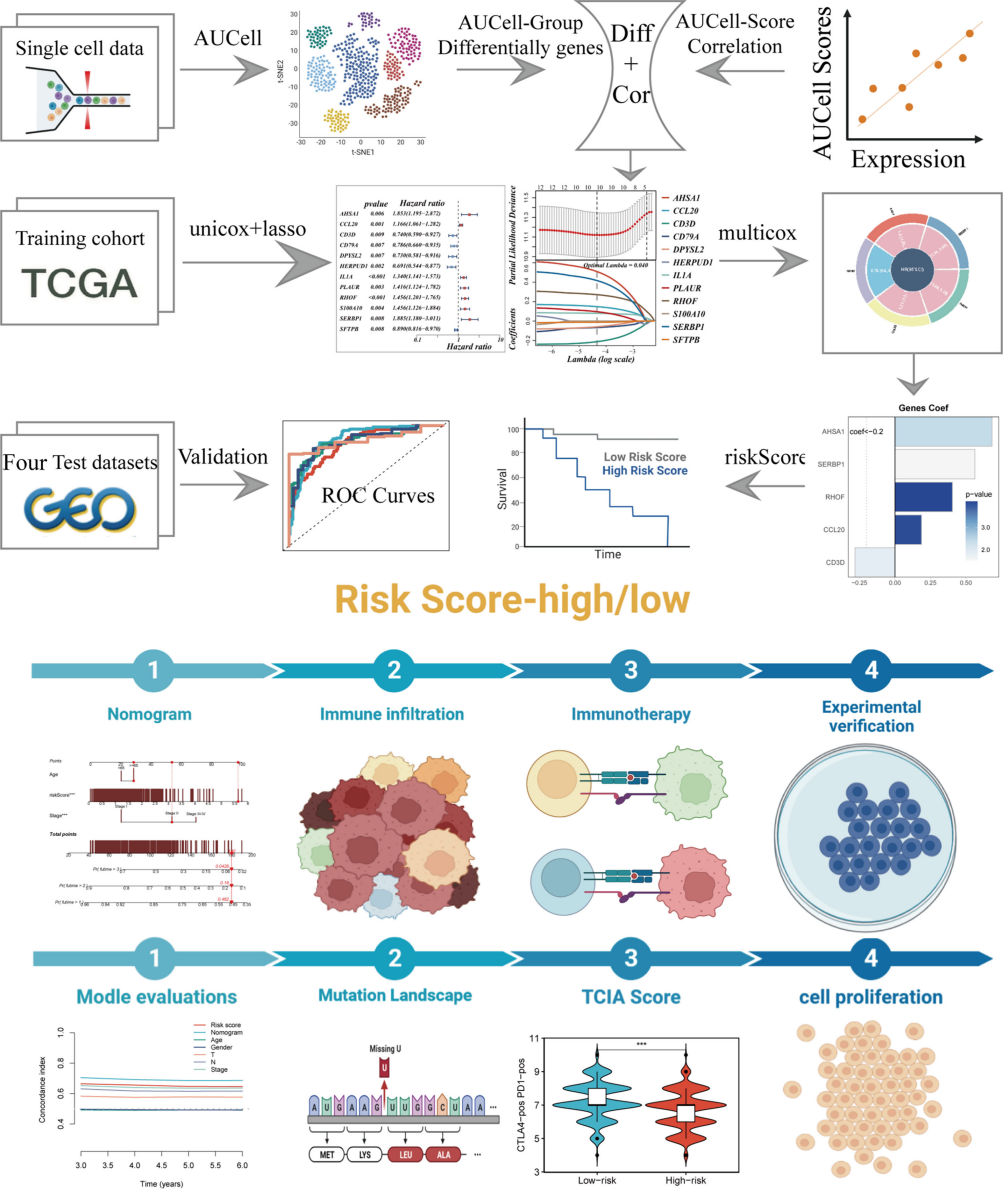
The workflow of the present study.

**Figure 2:**
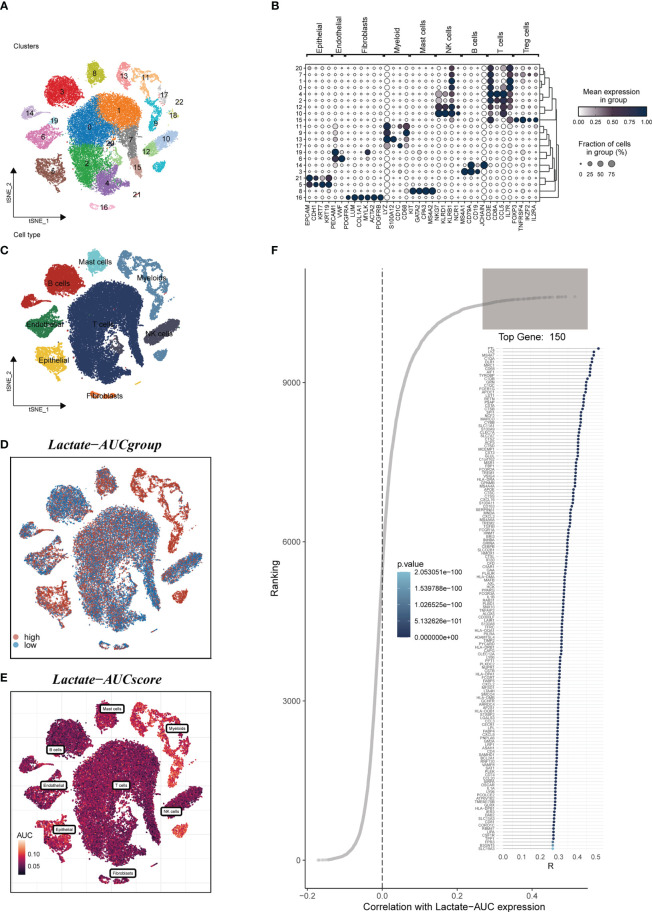
Annotation of single-cell data. **(A)** The t-SNE plot showed that all the cells in 22 clusters. **(B)** A bubble plot exhibited typical marker genes for each cell cluster. **(C)** The t-SNE map indicates that LUAD samples can be annotated as 8 cell types in the TME (different colors represent different cell types). **(D, E)** AUCell score and groups of LM activity in each cell. **(F)** Correlation analysis between LM-AUCell score and genes.

**Figure 1** depicted the flowchart for the study. A total of 46286 high-quality cells were deemed suitable for future study. The expression characteristics of each sample were shown in **Supplementary Figure S1A**. There was a statistically significant positive connection between sequencing depth and total intracellular sequences (R=0.94, **Supplementary Figure S1B**). The PCA reduction plot indicated no discernible differences in cell cycles (**Supplementary Figure S1C**). The study included 12 samples, and the cell distribution within each sample was mostly identical, indicating that there was no discernible batch impact between samples, which might be useful for future research (**Supplementary Figure S1D**). Following that, the dimensionality reduction methods, namely t-SNE, classified all cells into 22 clusters (**Figure 2A**). Bubble plots depicted the typical marker genes (19) of various cell types as well as the connection of distinct groups (**Figure 2B**). In **Figure 2C**, an t-SNE plot was used to depict the distribution of each cell population. Each cell’s LM activity was evaluated. AUC values were higher in cells that expressed more LMRGs, which were mostly orange-colored myeloid cells (**Figure 2D**). Based on the AUC score median values, all cells were assigned an AUC score for the LMRGs and divided into high- and low-LM-AUC groups (**Figure 2E**). Correlation study revealed that the genes most closely associated with LM activity (**Figure 2F**). The single-cell study yielded the 590 genes most linked with LM activity.

### 3.2 Construction and validation of the risk scoring

**Figure 3:**
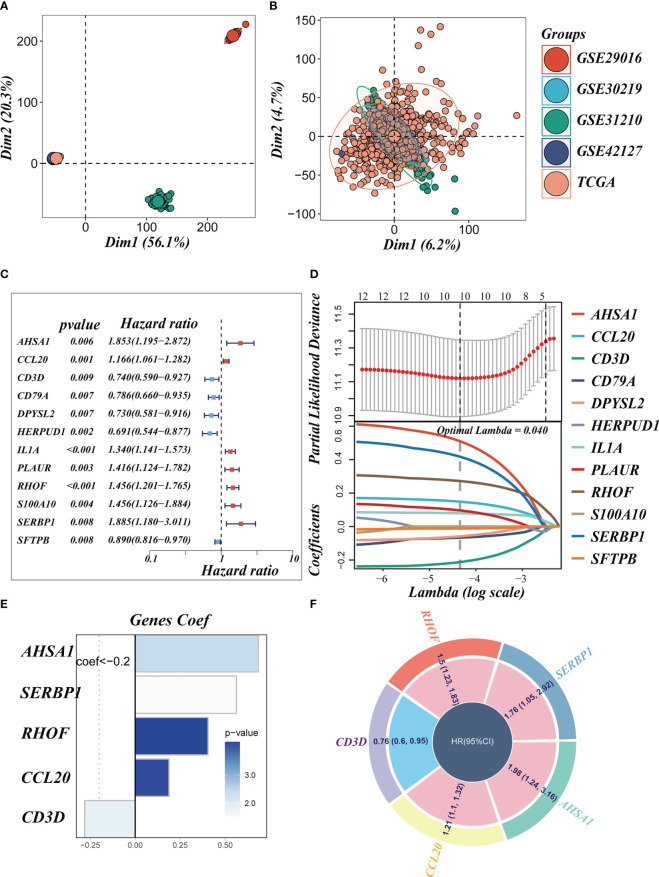
Construction of the signature. **(A, B)** PCA plots before and after removal of batch effects for 5 datasets. **(C)** A forest plot presents prognostic associated LMRGs. **(D)** Eleven prognostic LMRGs were included in the LASSO regression analysis to screen the most important model genes. **(E, F)** Coefficients for model genes as well as HR values for model genes.


We eliminated the batch effect from the GEO-obtained data for improved data consistency, and **Figures 3A, B** displayed the PCA plots before and after the batch effect was removed from the TCGA data, respectively. Following that, TCGA was separated into 6:4 training and validation sets, and univariate COX analysis was done, with the findings indicated by a forest plot (**Figure 3C**, P< 0.01), before lasso (**Figure 3D**) and multivariate COX regression analysis were used to create the risk model consisting of 5 genes. **Figure 3E** displayed the coefficients associated with each model gene from which the risk score was computed. The following was the formula:

$$risk\;score=\sum_{n=i}^{k}\left(Coef_{i}\mathrm{Exp}i\right)$$

The coefficient and expression of each model gene were represented by Coefi and Expi, respectively, and the risk score for each sample was determined using the above method. The circle diagram depicted the predictive HR value of five model genes, and it was obvious that AHSA1, SERBP1, RHOF, and CCL20 are at high risk. CD3D, on the other hand, had been demonstrated to be a low-risk gene (**Figure 3F**).

### 3.3 Survival analysis and model evaluation

**Figure 4:**
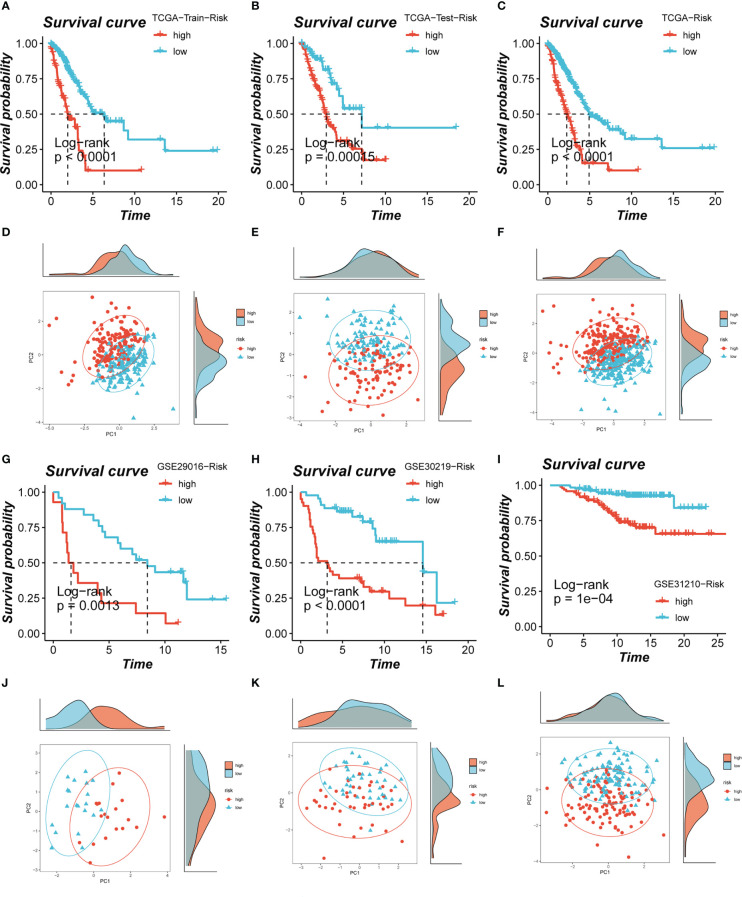
Assessment of risk models. **(A-C)** Kaplan-Meier survival analysis of signatures in the TCGA (train, test, and all set) datasets. **(D-F)** The PCA analysis was used to evaluate the distribution of the samples in the TCGA (train, test, and all set) datasets. **(G-I)** Kaplan-Meier survival analysis of signatures in the GEO (GSE29016, GSE30219, and GSE31210) datasets. **(J-L)** PCA analysis showed the distribution of samples in the GEO (GSE29016, GSE30219, and GSE31210) cohorts.

**Figure 5:**
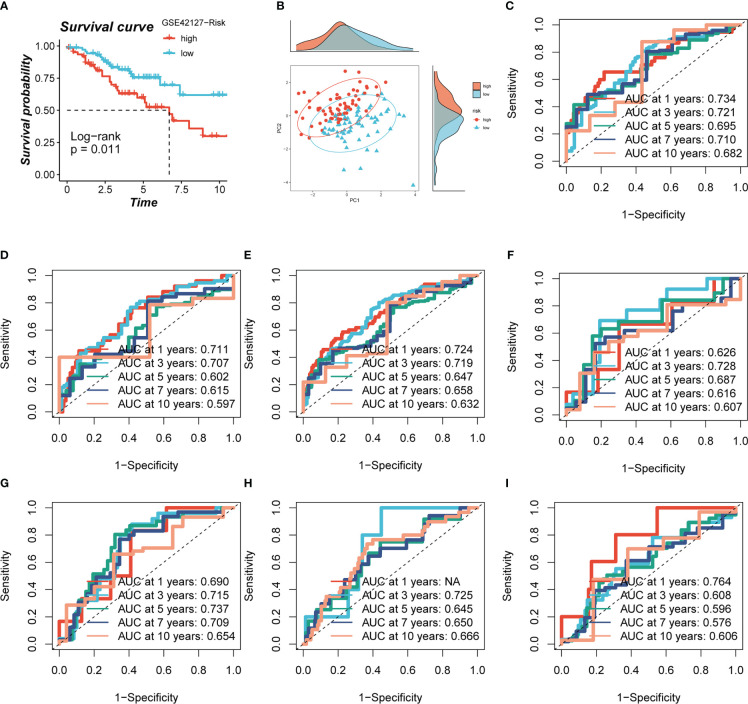
Evaluation of model. **(A, B)** Survival analysis revealed the survival significance of high and low risk scores in the GSE42127 cohort, and the sample distribution of high and low risk groups was shown in the PCA plot. **(C-I)** The ROC curve showed the survival accuracy of the model in TCGA (train, test, and all set) and GEO (GSE29016, GSE30219, GSE31210 and GSE42127) cohorts.

Based on median risk values, patients were split into high- and low-risk groups, and a survival analysis revealed a substantial OS difference for TCGA-LUAD patients (train set, test set, and all set, **Figures 4A–C**). Similarly, four GEO datasets also had statistically significant survival differences (P< 0.05; **Figures 4G–I**; **5A**). According to the expression levels of the model genes, PCA analysis was performed on all the samples from TCGA and GEO, and the results showed that the samples of the high- and low- risk groups could be clearly distributed into two clusters **Figures 4D–F, J–L** and **Figure 5B**. ROC analysis measured the discrimination of this signature, with 1-, 3-, 5-,7-, and10-year AUCs of 0.0.734, 0.721, 0.695, 0.710, and 0.682 in TCGA-train set; 0.711, 0.707, 0.602, 0.615, and 0.597 in TCGA-test set; 0.724, 0.719, 0.647, 0.658, and 0.632 in TCGA-all set; 0.626, 0.728, 0.687, 0.616, and 0.607 in GSE29016; 0.690, 0.715, 0.737, 0.709, and 0.654 in GSE30219; 0.725, 0.645, 0.650, and 0.666 in GSE31210 (LUAD-patients on survival less than 1 year were lacking); and 0.764, 0.608, 0.596, 0.576, and 0.6606 in GSE42127 (**Figures 5C-I**).

### 3.4 Construction and validation of nomogram

**Figure 6:**
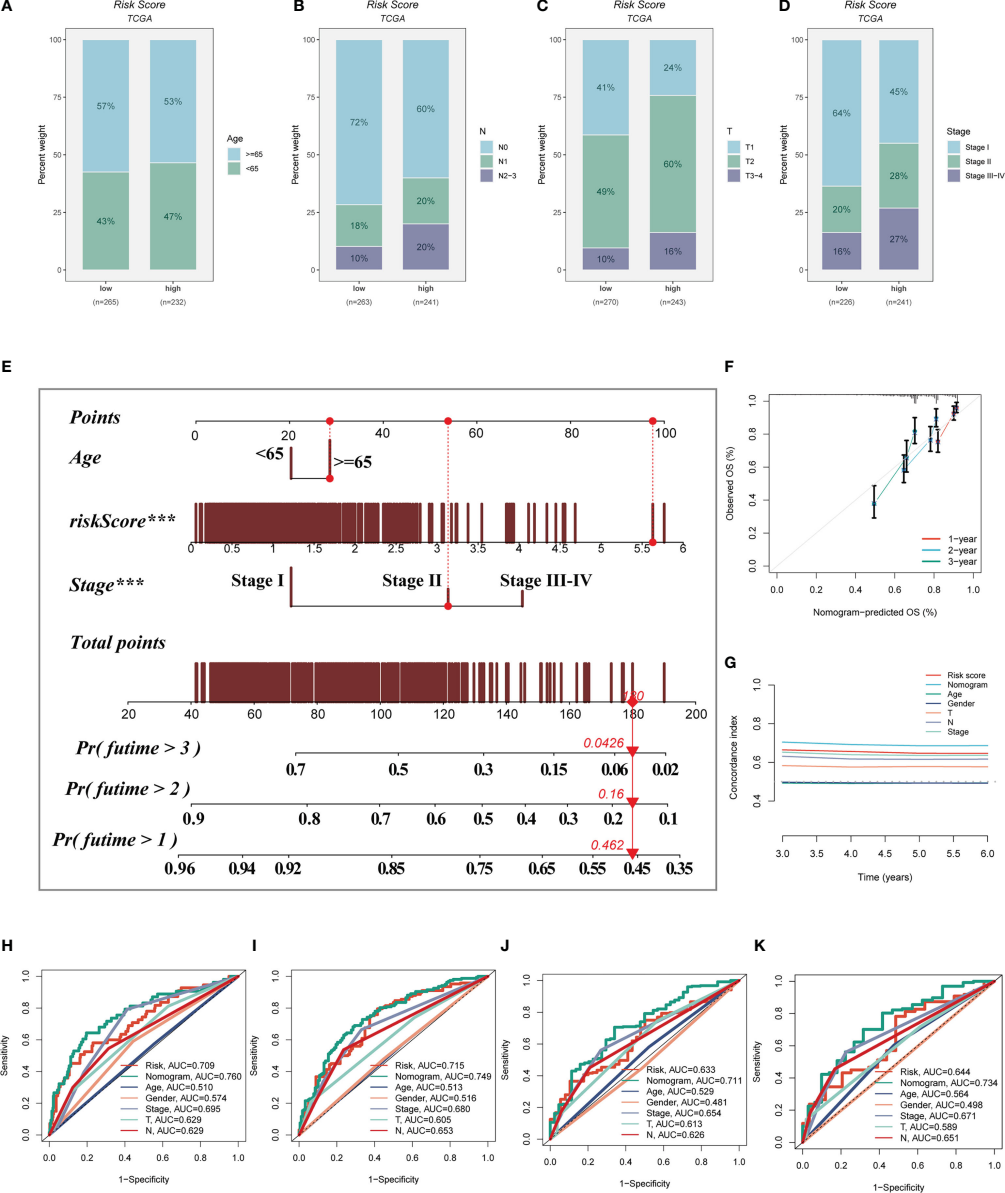
Building a more accurate nomogram. **(A-D)** The proportion of clinical characters (age, N stage, T stage, and clinical stage) in different risk groups. **(E)** Nomogram was constructed by combining clinical features with risk score. **(F)** The calibration plots test consistency between the actual OS rates and the predicted survival rates, with the 45°line representing the best prediction. **(G)** The C-index curves were used to evaluate the predictive performance of different clinical characteristics, nomogram scores and risk scores. **(H-K)** ROC curves for 1, 3, 5, and 7 years showed AUC values for various clinical factors, risk scores, and nomogram scores.

A heatmap was constructed to highlight the correlations between model genes and clinical characteristics. Some clinical factors (T stage, N stage, clinical stage, and survival status) differed significantly between the high- and low-risk groups (P< 0.05, **Supplementary Figure S2A**). The prevalence of different phases across groups was then compared and shown as a percentage bar plot. We observed that the high-risk group had worse T stage, N stage, and clinical stage (**Figures 6A-D**). Based on the TCGA-LUAD dataset, a predictive nomogram comprising risk score and clinicopathological parameters (age and clinical stage) was built to better predict prognosis (**Figure 6E**). Survival statuses at 1, 2, and 3 years were used as clinical outcome measures. The calibration plot revealed that this signature had outstanding prediction ability for 1-, 2-, and 3-year survival rates (**Figure 6F**). The C-index curves revealed that the nomogram outperforms the risk score and any other clinical measure in predicting prognosis (**Figure 6G**). The predictive ability of the nomogram score, risk score, and other clinical characteristics was also evaluated using ROC analysis. The AUC value of the nomogram score over one, three, five, and seven years was 0.760, 0.7749, 0.711, and 0.734, which were greater than risk scores and other clinical indicators (**Figures 6H–K**).

### 3.5 Mutational landscape

**Figure 7:**
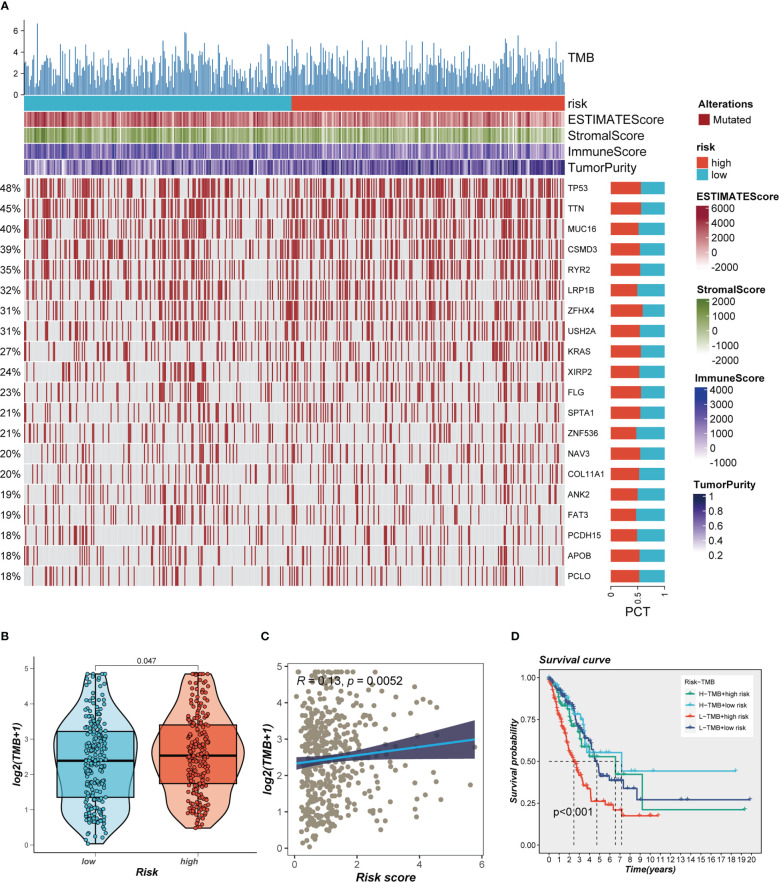
Landscape of LUAD sample mutation profiles. **(A)** Mutation landscape of the top 20 genes with mutation frequency in differential risk subgroups. **(B)** Comparison of tumor mutation burden (TMB) between different risk groups. **(C)** Correlation analysis between risk score and TMB. **(D)** Survival differences for four different subgroups (H-TMB+high-risk, H-TMB+low-risk, L-TMB+high-risk, and L-TMB+low-risk).

This was especially true for personalized cancer therapy, where mutations in certain genes play a crucial role. We studied the somatic mutation profiles of various risk categories. Statistics indicated that the high-risk group had a higher mutation frequency for the top 20 high-frequency mutated genes (**Figure 7A**), which included TP53, TTN, and CSMD3. **Figure 7B** indicated a significant difference in TMB between the high- and low-risk groups, with greater TMB in the high-risk group. Spearman correlation analysis was utilized to study the association between risk score and TMB, and a significant positive correlation was obtained (R = 0.12, P< 0.001, **Figure 7C**). We then divided patients into four groups (H-TMB+high-risk, H-TMB+low-risk, L-TMB+high-risk, and L-TMB+low-risk) based on median TMB values and median risk values; the results showed that LUAD patients with H-TMB+low-risk had the best prognosis, and LUAD patients with L-TMB+high-risk had the worst prognosis (**Figure 7D**).

### 3.6 Differences in the immune microenvironment and immunotherapy response

**Figure 8:**
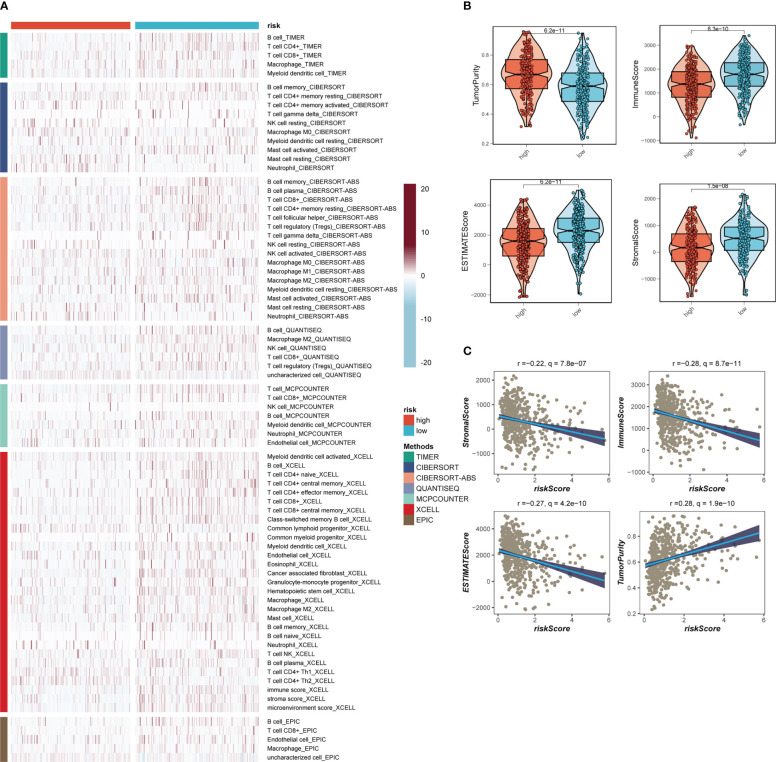
Analysis of immune infiltration. **(A)** Seven algorithms assess differences in immune infiltration status between different risk groups. **(B)** The violin plot demonstrated the difference in Stromal Score, Immune Score, ESTIMATE Score, and tumor purity calculated using the ESTIMATE algorithm between the two risk subgroups. **(C)** The correlations in Stromal Score, Immune Score, ESTIMATE Score, and tumor purity calculated using the ESTIMATE algorithm between the two risk subgroups.

**Figure 9:**
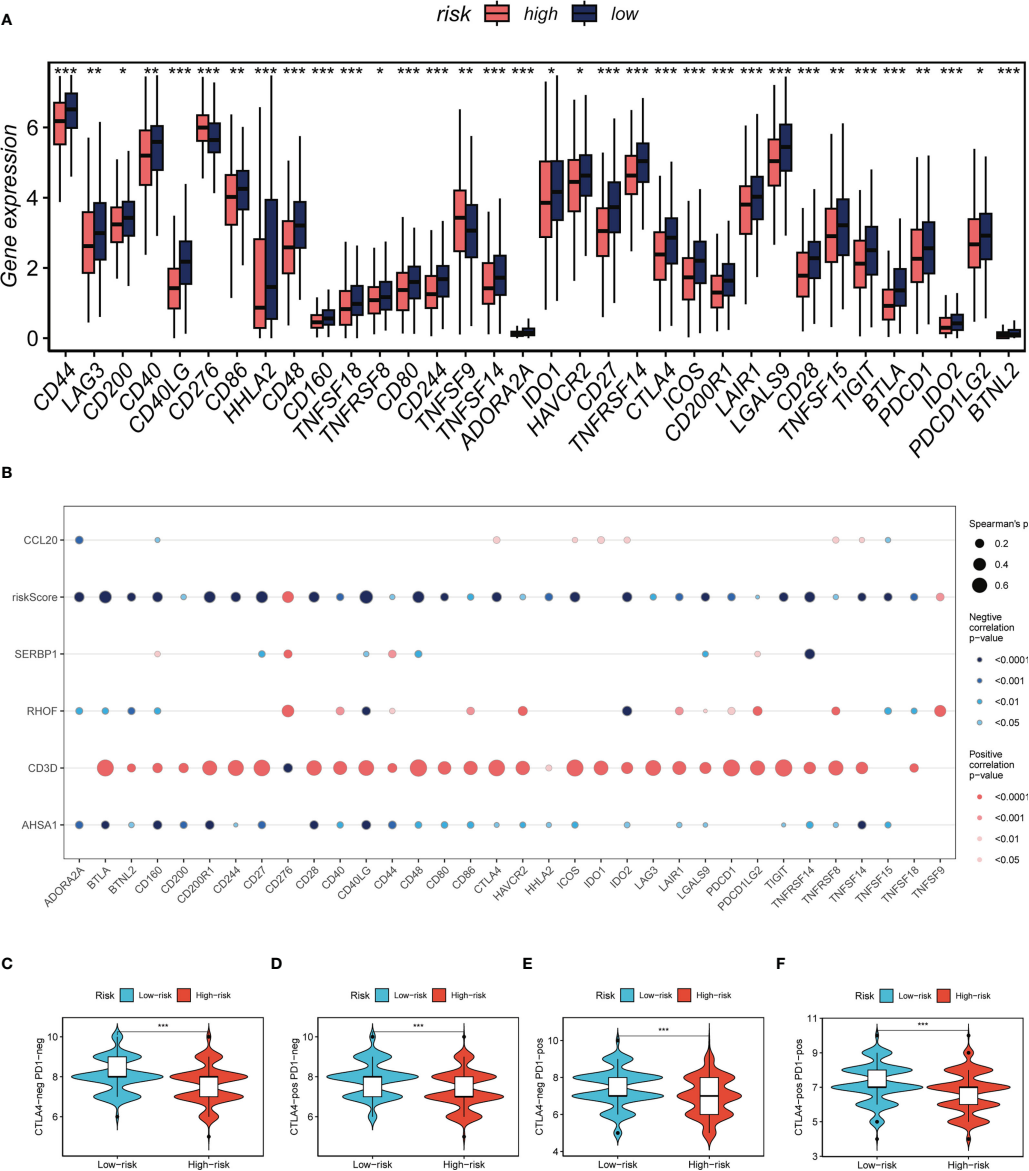
Immune checkpoint and TCIA analysis. **(A)** A box plot showed that differences in immune checkpoint gene expression between high- and low-risk groups. **(B)** Correlation between model genes and immune checkpoint. **(C-F)** The low-risk group has significantly greater IPS, IPS-CTLA4-neg-PD-1-neg, IPS-CTLA4-pos-PD-1-neg, IPS-CTLA4-neg-PD-1-pos, and IPS-CTLA4-pos-PD-1-pos. Note: *P< 0.05, **P< 0.01, ***P< 0.001.

Seven separate algorithms indicated that tumors at low risk had greater immune cell infiltration, such as T cells, B cells, NK cells, and activated Mast cells as illustrated in **Figure 8A**. The ESTIMATE approach was used to analyze the amount of immune infiltration in the various risk groups, and **Figure 8B** similarly confirmed the prior study, with the low-risk group having greater stromal, immunological, and ESTIMATE scores than the other groups (stromal score combined with immune score). Spearman correlation analysis was utilized to evaluate the link between risk score and the score of immune infiltration. The risk scores were favorably connected with stromal (R = -0.22, FDR< 0.001), immune (R = -0.28, FDR< 0.001), and ESTIMATE (R = -0.27, FDR< 0.001) scores, and negatively correlated with tumor purity (R = -0.28, FDR< 0.001, **Figure 8C**). The risk score was correlated with the degree of immune cell infiltration and the quantity of each component in the TME, according to the data. Depending on the degree of immune infiltration, disease progression and immunotherapeutic efficacy may differ. Given the above results, we investigated whether the prognostic model might predict LUAD patients’ reaction to ICIs. First, we examined the relation between risk score and commonly identified immunotherapy biomarkers in the TCGA-LUAD cohort. It demonstrated that practically all ICGs, including as PD-1, TIGIT, and CTLA4, were all substantially expressed in the high-risk group (**Figure 9A**). The correlations between modeling genes, risk scores, and ICGs were further analyzed and shown in the bubble plot (**Figure 9B**), with blue representing negative correlations and orange representing positive correlations, with bigger bubbles and deeper hues suggesting a stronger link. The IPS can help you locate persons who possibly benefit from immunotherapy. It was hypothesized that tumor samples from these individuals would have a positive immune response to PD-1/PD-L1 or CTLA4 inhibitors, or both (**Figures 9C-F**). Patients in the group with the lowest risk had much higher IPS scores, indicating that they would benefit the most from this kind of immunotherapy.

### 3.7 Functional enrichment analysis

**Figure 10:**
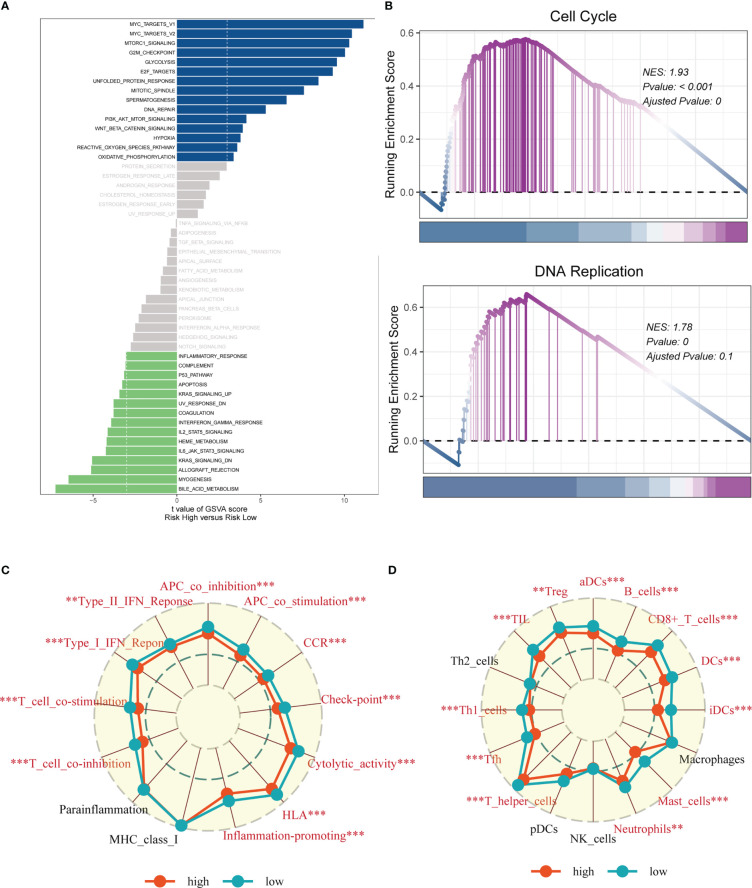
Enrichment analysis. **(A)** GSVA analysis revealed pathways enriched in the 50 hallmark gene sets for the high- and low- risk groups. **(B)** GSEA showed pathway differences between high- and low-risk groups. **(C, D)** The ssGSEA algorithm was employed to quantify the immune cell infiltration and immune function between the high-risk and low-risk groups. Note: **P < 0.01, ***P < 0.001.

In order to investigate the underlying process that may lead to a poor prognosis for high-risk LUAD patients, an analysis of hallmark pathway gene profiles was conducted, revealing distinct characteristics between high- and low-risk groups. A direct comparison between the Risk-High and Risk-Low groups showed that the top five enriched signatures in the high-risk group were MYC target v1, MYC target v2, mTORC1 signaling, G2M checkpoint, and Glycolysis (**Figure 10A**). GSEA enrichment analysis also indicated that the high-risk group had significantly enriched Cell Cycle (NES = 1.93, p< 0.001) and DNA Replication (NES = 1.78, p = 0.000) (**Figure 10B**). The ssGSEA algorithm was employed to examine differences in immune status across distinct risk groups. Low-risk LUAD patients showed increased infiltration of various immune cells, including Active dendritic cells (aDCs), B cells, CD8+ T cells, Dendritic cells (DCs), Immature dendritic cells (iDCs), Mast cells, neutrophils, T helper cell, Tumor-infiltrating lymphocytes (TILs), and Regulatory T cells (Treg), in their tumor microenvironment (TME). Furthermore, almost all immune-related pathways were significantly expressed in the low-risk group (**Figures 10C, D**).

### 3.8 Experimental verification

**Figure 11:**
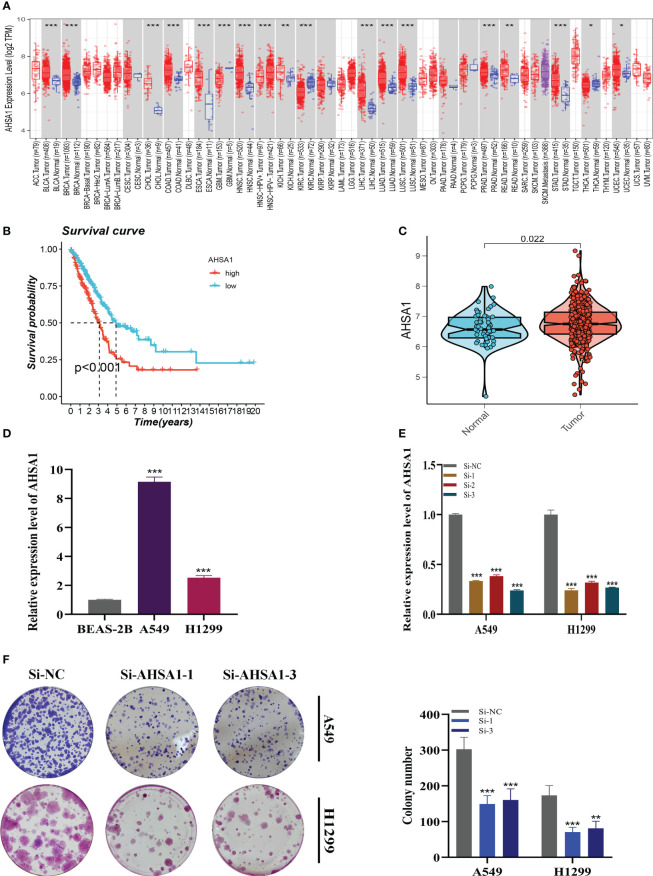
Cell Experiment. **(A)** The expression of AHSA1 in pan-cancer tissues was analyzed using the TIMER database. **(B)** Prognosis was evaluated by performing survival analysis on the effect of AHSA1 expression. **(C)** TCGA database analysis revealed a difference in AHSA1 expression between normal samples and tumor samples. **(D)** To assess AHSA1 expression, qRT-PCR was performed on both normal cells and LUAD cell lines. **(E)** The level of AHSA1 expression was evaluated 5 days after transfection using qRT-PCR, and significant reduction in AHSA1 expression (P< 0.001) was observed with siRNA sequences. **(F)** The number of colonies was significantly reduced in cells with reduced AHSA1 expression compared to the NC group, as shown by the colony formation assay. Note: *P < 0.05, **P < 0.01, ***P < 0.001.

**Figure 12:**
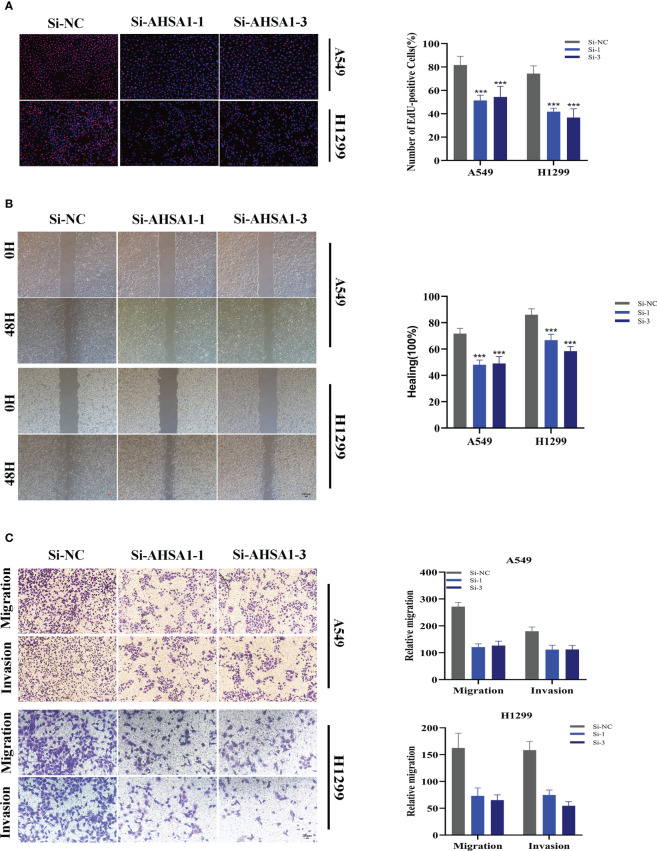
Related experiments for AHSA1. **(A)** EdU staining assay indicated that downregulation of AHSA1 expression repressed cell proliferation in LUAD cell lines. **(B)** Scratch-wound healing assay depicted that a significantly slower wound healing rate was observed in cells with a decreased expression of AHSA1. **(C)** Transwell assay showed that downregulation of AHSA1 expression inhibited the migration and invasion capacity of LUAD cells. To demonstrate the accuracy and reproducibility of the results, all experiments were repeated in two LUAD (A549, H1299) cell lines and all data were presented as the means ± SD of three independent experiments. Note: ***P < 0.001.

A pan-cancer study of AHSA1 expression levels demonstrated that AHSA1 was substantially expressed in LUAD compared to normal tissue (**Figure 11A**). **Figures 11B, C** demonstrated that AHSA1 was substantially expressed in tumor groups and that patients with high AHSA1 expression in the TCGA database had a worse prognosis. In accordance with our earlier findings, AHSA1 was expressed at a greater level in LUAD cell lines (**Figure 11D**). Then, five days after transfection, we quantified the amount of AHSA1 expression in A549 and H1299 cell lines by qRT-PCR to determine the efficiency of siRNA-mediated AHSA1 knockdown (**Figure 11E**). According to research on clonal formation, AHSA1 knockdown inhibits the capacity of LUAD cells to produce clones (**Figure 11F**). Then, EdU tests were conducted to investigate whether knockdown of AHSA1 affected the proliferative capacity of LUAD cells. Lower AHSA1 expression decreased the proliferation of A549 and H1299 cells relative to the control group (**Figure 12A**), indicating that AHSA1 may play a role in the proliferation of LUAD cell lines. According to these results, AHSA1 knockdown inhibited the proliferation of LUAD cells. The investigation on wound healing demonstrated that AHSA1 knockdown dramatically decreased LUAD cell migration and invasion (**Figure 12B**). The trans-well experiment demonstrated that LUAD cells transfected with si- AHSA1 exhibited a reduced capacity for migration and invasion, which was consistent with the wound healing assay outcomes (**Figure 12C**). All experimental investigations demonstrated that AHSA1 was a tumor-promoting oncogene in tumor development and progression and acted as a pro-oncogenic regulator in LUAD.

## 4 Discussion

LC remains one of the most prevalent malignant tumors and the greatest contributor to cancer-specific death worldwide. Despite significant improvements have been made in diagnostic techniques and treatment schedules of NSCLC, the 5-year overall survival rate remains poor. At present, lactic acid, the byproduct of glycolysis, was repeatedly confirmed could promote malignant cell proliferation and induce immunosuppressive microenvironment. It acts as a mediator between intrinsic metabolism and immunosuppression. In recent years, researchers found that reducing the concentration of lactate might be a promising therapeutic strategy. Hence, lactate-related genes have the potential to act as novel molecular biomarkers and therapeutic targets. In the present study, we explored an original diagnostic signature and prognostic scoring system based on LRMGs, bringing prospect for reversing immune resistance and improving prognosis of patients. Numerous groundbreaking research demonstrated the potential of the lactic acid-induced immunosuppressive milieu and its role in the promotion of tumors. As far as we are aware, LUAD does not have a lactate-related prognostic grading system.

We conducted scRNA-seq on 12 LUAD samples in this study and identified eight distinct cell types. LM activity was evaluated using the LM gene set obtained from the GeneCards database, and myeloid and epithelial cells were found to exhibit the highest levels of LM activity, suggesting that LM may play a role in regulating these cells and influencing carcinogenesis and development. Key genes that regulate LM activity were then investigated, and prognostic models were constructed using Cox and lasso regression. The high-risk group was found to have a worse prognosis, and a signature derived from this analysis demonstrated good accuracy and stable performance across four public GEO datasets. We also integrated clinical information to develop a nomogram, which showed better performance in predicting survival than risk scores and other clinical characteristics. While previous studies have suggested a link between genetic modifications and the generation of neoantigens and potential immunotherapeutic advantages (20), our findings showed that patients in the low-risk group had fewer TMB, while patients in the high-risk group had more mutations in high-frequency genes. We further categorized the patients into four groups based on TMB and risk status, and the H-TMB+low-risk group had the best prognosis, providing potential clinical implications for prognostic assessment.

The immune microenvironment is composed of a variety of cellular components including extracellular matrix, epithelial cells, blood vessels and tumor-infiltrating lymphocytes, which may accelerate tumor destruction, enhance tumor invasiveness, and improve antitherapeutic response (21). To further understand how TME effects tumor prognosis, we examined immune cell infiltration in high- and low-risk LUAD patients. Seven algorithms were used to quantify immune cell infiltration in various risk categories, and the results revealed that tumors in the low-risk group had more immune cell infiltration. The ESTIMATE approach also revealed that low-risk samples had more immune cell infiltration, and the risk score was inversely connected to the stromal, immune, and ESTIMATE scores (FDR< 0.001). Furthermore, we discovered that the majority of the known ICGs were expressed at a greater level in the low-risk group, and the correlation analysis revealed that the risk scores were strongly negatively linked with the majority of the immunological checkpoint genes. TCIA was utilized to investigate the effects of PD-1 and CTLA-4 treatment in order to better understand the variations in immunotherapy effectiveness among risk groups. Because their IPS score was substantially higher than that of the high-risk group, the findings suggested that LUAD patients in the low-risk group would benefit more from immunotherapy.

GSEA results show that Cell Cycle and DNA Replication were mainly enriched in the high-risk group. Tumor is a kind of disease in which cell cycle regulation mechanism is destroyed. In the whole monitoring system of cell cycle progression, cell cycle detection sites play a core role function. DNA replication is an important part of the cell cycle, dysregulation of which is also one of the significant factors leading to tumorigenesis and tumor proliferation. Currently, cell cycle checkpoint kinase inhibitors are utilized therapeutically and are successful in LC. These inhibitors induce cell death and cell cycle arrest, therefore reversing the acquired drug resistance induced by cell cycle disorder (22).

Interestingly, in TCGA database, AHSA1 was highly expressed in tumor groups, and LUAD patients with high-expression AHSA1 had poor prognosis. In order to understand the underlying mechanism, we conducted a series of experiments. According to the results, knocking down AHSA1 significantly decreased cell invasion, migration, and proliferation in LUAD cell lines.

The current research has certain problems. To begin, this signature was built utilizing publicly accessible datasets. Large-scale prospective clinical investigations are required to verify the prognostic potential. In conclusion, we constructed an LM-related signature, which can predict the prognosis and immunotherapy of LUAD patients, and our findings can provide help for the clinical treatment of LUAD.

## Data availability statement

The datasets presented in this study can be found in online repositories. The names of the repository/repositories and accession number(s) can be found in the article/**Supplementary Material**.

## Author contributions

PZ, SP, ZG, and QR contributed conception and design of the study. JX, HL and WW collected the data. PZ and SP performed the statistical analysis. PZ, SP, and ZG wrote the first draft of the manuscript. JX, HL, and WW gave the final approval of the version to be submitted. All authors contributed to the article and approved the submitted version. 
