# Supplementary figures and images for: The integrated single-cell analysis developed a lactate metabolism-driven signature to improve outcomes and immunotherapy in lung adenocarcinoma
Source: Front Endocrinol (Lausanne). 2023 Mar 22;14:1154410. doi: 10.3389/fendo.2023.1154410 (PMC10073691; doi:10.3389/fendo.2023.1154410)

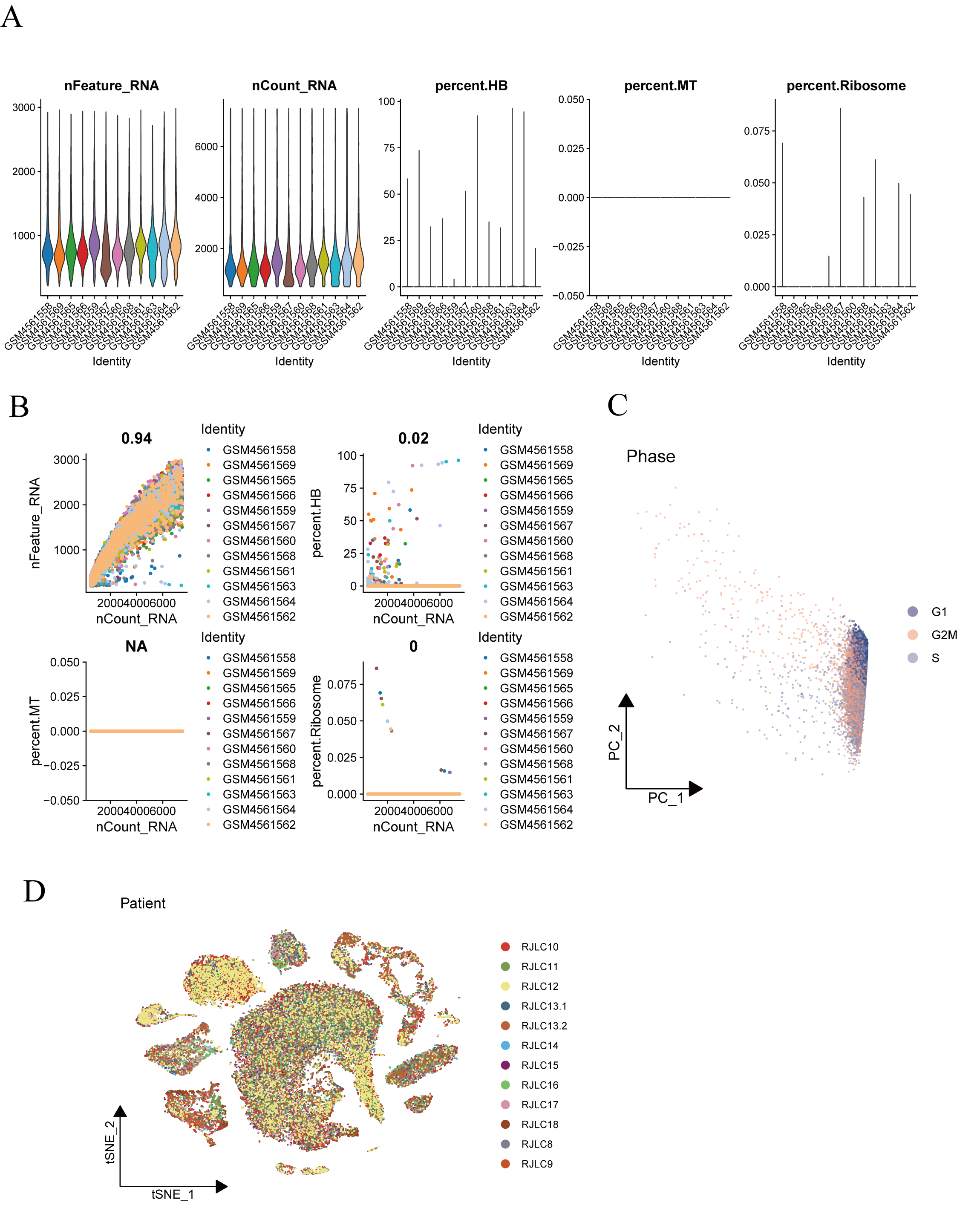

Supplement: Supplementary file 1 [file Image_1.tif]

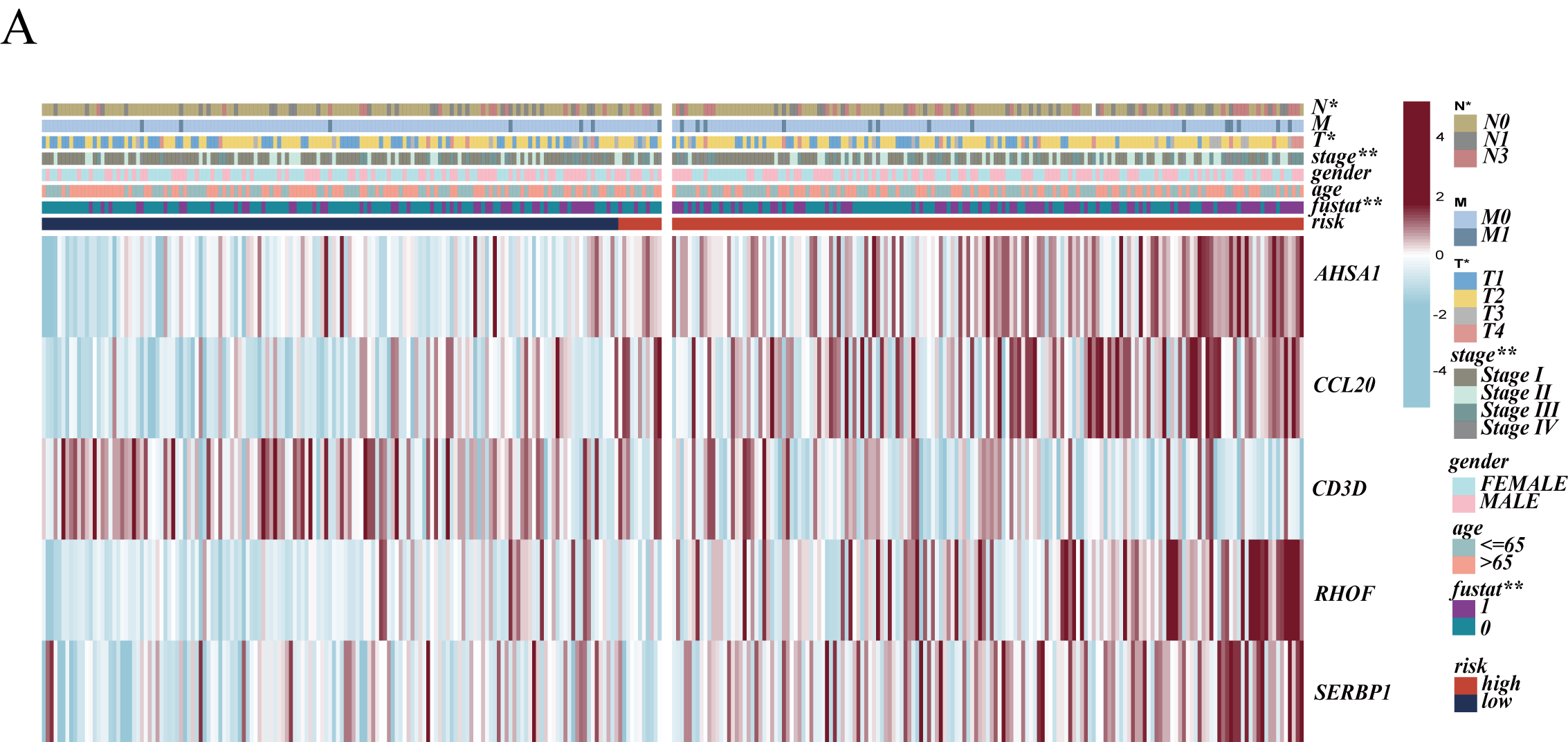

Supplement: Supplementary file 2 [file Image_2.tif]
