# Supplementary material for: The integrated single-cell analysis developed a lactate metabolism-driven signature to improve outcomes and immunotherapy in lung adenocarcinoma
Source: Front Endocrinol (Lausanne). 2023 Mar 22;14:1154410. doi: 10.3389/fendo.2023.1154410 (PMC10073691; doi:10.3389/fendo.2023.1154410)
Supplement: Supplementary file 3 [file Table_1.docx]

| **Oligonucleotides** | **Nucleotide sequence (5'-3')** |
| --- | --- |
| **siRNA** |  |
| Scramble control | CCTAAGGTTAAGTCGCCCTCG |
| Si- AHSA1-1 | GCATGATCTTACCTACAAT |
| Si- AHSA1-2 | CCATCACCTTGACCTTCAT |
|  |  |
| **Primer** |  |
| GAPDH | GGCCTCCAAGGAGTAAGACC (forward) |
|  | AGGGGAGATTCAGTGTGGTG (reverse) |
| AHSA1 | GGCACTA AGCGGTCCTGAG (forward) |
|  | CTCCACTTCATCCACGCTGT (reverse) |
|  |  |

**Table S1. Oligonucleotides used in research**
